# Facial Neuralgia and Allied Neurosis

**Published:** 1890-04

**Authors:** 


### Facial Neuralgia and Allied Neurosis.

Mr. Leslie, M.B. (*Edinburgh Med. Journal*), states that about three months ago he found he was able to arrest a very severe attack of supra-orbital neuralgia in a very short time by applying common salt to the nasal mucous membrane. During the last three months the author has been following up this method of treatment in cases of neuralgic headache, faceache, toothache, earache, etc., and has found that in nearly every case a rapid cure is obtained. Common table salt may be used by the patient as snuff, a pinch being taken into the nostril of the affected side; but the best results have been obtained when the salt has been applied by means of an insufflator. In practice, the author charges a small insufflator with about four grains, and the patient is told to draw air up the nostril while the contents of the insufflator are injected. The application produces little pain or discomfort, and relief is speedy. The stimulation by chloride of sodium appears to induce in the nasal branches of the fifth nerve a form of nerve motion, which causes reflex inhibition of the pathological process in the nerves affected, inhibits the abnormal form of nerve-energy, of which the expression is pain, and replaces it by the normal form, of which the expression is not pain. Although a single application usually suffices for the immediate inhibition of neuralgia, especialy when it is recent and localized in one branch of the fifth nerve, in other cases where the disease has been of long standing and of extensive distribution, it may be necessary to repeat the insufflation every half minute for about five minutes.—*London Med. Recorder*.

ENGLISH newspapers are earnestly condemning Egyptian tobacco and the doctored cigarettes made therefrom. It is contended that a large number of cases of mouth-disease have resulted from the use of these cigarettes, and the discussion, which is becoming hot and interesting, will very likely result in serious injury to the eastern tobacco trade.—*San. News*.

